# Failure to respond to the surface of *Plasmodium falciparum* infected erythrocytes predicts susceptibility to clinical malaria amongst African children

**DOI:** 10.1016/j.ijpara.2008.03.009

**Published:** 2008-10

**Authors:** C.L. Mackintosh, T. Mwangi, S.M. Kinyanjui, M. Mosobo, R. Pinches, T.N. Williams, C.I. Newbold, K. Marsh

**Affiliations:** a Kenya Medical Research Institute, Centre for Geographic Medicine Research Coast (KEMRI-CGMRC), Kilifi District Hospital, Kilifi, Kenya; b Molecular Parasitology Group, Weatherall Institute of Molecular Medicine, John Radcliffe Hospital, Oxford OX3 9DS, UK; c Nuffield Department of Medicine, John Radcliffe Hospital, Oxford OX3 9DS, UK; d Department of Paediatrics, John Radcliffe Hospital, Oxford OX3 9DS, UK

**Keywords:** *Plasmodium falciparum*, Malaria, Antibody responses, Susceptibility, Immunology, Epidemiology, Children, Sub-Saharan Africa

## Abstract

Following infection with *Plasmodium falciparum* malaria, children in endemic areas develop antibodies specific to antigens on the parasite-infected red cell surface of the infecting isolate, antibodies associated with protection against subsequent infection with that isolate. In some circumstances induction of antibodies to heterologous parasite isolates also occurs and this has been suggested as evidence for cross-reactivity of responses against the erythrocyte surface. The role of these relatively cross-reactive antibodies in protection from clinical malaria is currently unknown. We studied the incidence of clinical malaria amongst children living on the coast of Kenya through one high transmission season. By categorising individuals according to their pre-season parasite status and antibody response to the surface of erythrocytes infected with four parasite isolates we were able to identify a group of children, those who failed to make a concomitant antibody response in the presence of an asymptomatic parasitaemia, at increased susceptibility to clinical malaria in the subsequent 6 months. The fact that this susceptible group was identified regardless of the parasite isolate tested infers a cross-reactive or conserved target is present on the surface of infected erythrocytes. Identification of this target will significantly aid understanding of naturally acquired immunity to clinical malaria amongst children in endemic areas.

## 1 Introduction

Non-sterile immunity to *Plasmodium falciparum* malaria is acquired by individuals living in endemic areas, enabling them to maintain infections without the associated morbidity and mortality experienced by non-immune individuals. Evidence from the passive transfer of antibodies from immune to non-immune individuals suggests this immunity is, at least in part, antibody mediated (Cohen et al., 1961; Edozien et al., 1962). Humans exposed to malaria mount antibody responses to a wide range of parasite antigens, the majority of which are unlikely to be associated with protective immunity. The antibody response to variant parasite antigens expressed on the surface of the infected erythrocyte (VSA) have, however, been associated with protection and are specific to the infecting isolate following any single episode (Marsh et al., 1989; Bull et al., 1998; Giha et al., 2000; Chattopadhyay et al., 2003). Among children exposed to multiple infections, it has been proposed that clinical malaria may be, in part, a consequence of gaps in their anti-VSA antibody repertoire (Bull et al., 1998).

The induction of antibodies during an acute episode to the infected red cell surface of parasite isolates apparently not involved in that episode (heterologous parasites) has been suggested as evidence for some cross-reactivity in these responses (Giha et al., 1998; Chattopadhyay et al., 2003). However, the role of such heterologous responses in protection from clinical malaria is not clear. Studies from disparate regions across Africa measuring responses against locally obtained clinical isolates, isolates taken from individuals resident in geographically distinct regions and various laboratory reference lines have yielded conflicting results, suggesting that antibody responses to some parasites but not others are associated with future protection from clinical malaria (Marsh et al., 1989; Bull et al., 1998, 2002; Giha et al., 2000; Dodoo et al., 2001). In none of these studies, however, was parasite status at the time of serum collection taken into account.

Several studies have demonstrated an association between *P. falciparum* infection and enhanced anti-erythrocyte surface antibody responses to a range of isolates (Iqbal et al., 1993; Giha et al., 1999a,b; Ofori et al., 2002). In support of this, more recent data from our group demonstrated that the proportion of isolates recognised was strikingly higher amongst children with a microscopically detectable parasitaemia at the time of assay compared with those without and that this association was not just due to cumulative exposure. Rather, it suggests that the presence of parasites reveals short-lived, more cross-reactive responses (Bull et al., 2002; Kinyanjui et al., 2004b). The presence of parasites at the time of serum collection not only leads to increased antibody recognition but also modifies the likelihood that this measured response will be associated with protection from both severe and mild clinical malaria (Bull et al., 2002; Kinyanjui et al., 2004b; Polley et al., 2004; Osier et al., 2007). The precise target on the infected erythrocyte surface for these short-lived responses is currently unknown.

Using a longitudinal study design, we have examined the relationship between antibodies to antigens on the surface of erythrocytes infected with *P. falciparum* and subsequent protection from mild clinical malaria. We have compared responses to three laboratory parasite lines, including two different phenotypes of the same isolate, with responses to a locally acquired clinical isolate, and by factoring into the analysis the interplay between antibody responses and the presence of parasites, we demonstrate that failure to mount an antibody response to the surface of the erythrocyte infected with any isolate tested predicts subsequent susceptibility to malaria amongst asymptomatically parasitised children. We also observe a strong correlation in individual antibody responses to each parasite tested, suggesting a more conserved target on the infected erythrocyte surface for these responses.

## 2 Materials and methods

### 2.1 Study population

This work was carried out at the Kenya Medical Research Institute (KEMRI) Centre for Geographic Medicine Research Coast (CGMRC) situated at Kilifi District Hospital, 50 km north of Mombasa on the coast of Kenya. The hospital serves approximately 240,000 people. Individuals investigated during these immuno-epidemiological studies were resident in Kilifi District, in an area called Chonyi. This study site has been described in detail elsewhere (Mwangi et al., 2005). Inhabitants of this area are predominantly Mijikenda. Residents of Chonyi have an estimated 50 bites/person/year (Mbogo et al., 2003). The overall annual incidence of clinical malaria has been reported as 0.55 episodes/person/year (Mwangi et al., 2005).

### 2.2 Sample collection

Sera collection and active surveillance were conducted as part of a study examining the clinical epidemiology of malaria under differing transmission conditions (Mwangi et al., 2005). In brief, after informed consent was obtained from parents or guardians, serum was collected in October 2000 from 272 children aged between 6 months and 10 years (Table 1). A blood slide was prepared for every individual in the cohort at the time of serum collection in order to define their pre-clinical surveillance status as parasite-positive or -negative and the cohort was followed for evidence of malaria by weekly active surveillance for fever. Malaria was defined as a febrile episode when individuals presented with an axillary temperature greater than 37.5 °C and a parasitaemia greater than 2500 parasites/μl above 1 year of age, and fever plus any parasitaemia below 1 year of age. These have been determined to be sensitive and specific malaria case definitions in this study community (Mwangi et al., 2005). Twenty non-malaria exposed control sera were collected from Oxford, United Kingdom.

Sera from a further cohort of children were analysed in order to examine antibody responses to a heterologous isolate in a known susceptible group of children. This cohort was recruited as part of an investigation into the kinetics of the antibody response to acute malaria (Kinyanjui et al., 2003). In brief, sera from 39 children with a mean age of 29.5 months (95% Confidence Interval (CI) 20.3–38.7) resident in Kilifi District were collected at presentation to Kilifi District Hospital with a primary diagnosis of *P. falciparum* malaria. Children were followed up at 1, 2, 3 and 6 weeks after treatment and further serum samples obtained. The precise details of recruitment and sampling of this cohort have been published elsewhere (Kinyanjui et al., 2003).

### 2.3 Parasites

Initially, antibody responses were measured to a laboratory clone of parasite, A4. This particular parasite was chosen for a number of reasons. There exists a monoclonal antibody, BC6, which allows in vitro selection of A4 parasites specifically expressing A4 *Pf*EMP1(Roberts et al., 1992). A4 *Pf*EMP1 has a well-described and classified sequence already published with established domain boundaries and it displays a common cytoadherent phenotype, binding to both CD36 and ICAM-1 (Smith et al., 2000). The parasite clone A4 having undergone prior selection with BC6 is denoted A4U.

Sera from 140 individuals randomly selected from the initial cohort of 272 were subsequently tested for recognition of two further laboratory clones of parasite, A4 40-cycle (parasites of the A4 lineage which have been left in culture for 40 cycles with no selection, thus expressing on the erythrocyte surface a more heterogeneous group of *var* genes), 3D7 and one clinical isolate, P1, obtained from a 5-year-old child admitted to Kilifi District Hospital with moderately severe malaria (Table 2).

Sera from the cohort of children recruited on presentation to hospital were tested for recognition of A4U at presentation and subsequently at each follow-up time point.

### 2.4 Flow cytometry

Using parasite clones A4U, A4 40-cycle, 3D7 and the clinical isolate, denoted P1, cryo-preserved trophozoite-infected erythrocytes at between 1% and 5% parasitaemia were thawed through the sequential restoration of isotonicity (Kinyanjui et al., 2004a). They were washed twice in RPMI and the pellet was resuspended at 1% haematocrit in 0.1% BSA/PBS. One microlitre of human serum was pipetted into separate wells of a 96-well U-bottomed plate (Nunc Technology) and 9 μl of the infected erythrocyte cell suspension was added to each well, giving a final test serum concentration of 1:10. The reaction mixture was incubated at room temperature for 1 h, following which the cells were spin washed three times with 0.1% BSA/PBS. The cells were then resuspended in 25 μl of 0.1% BSA/PBS containing the secondary antibody, rabbit anti-human IgG at a concentration of 1:100. Again the reaction mixture was incubated for 1 h at room temperature after which a further three washes were performed as before. Finally 25 μl of 0.1% BSA/PBS containing a 1:100 dilution of swine anti-rabbit IgG coupled to FITC and 10 μg/ml of ethidium bromide was added to each well. A further incubation at room temperature in darkness for 1 h was done after which, following a further series of washes, at least 1000 infected erythrocytes were counted on an EPIC/XL flow cytometer (Coulter-Electronics, UK).

Reactivity against the infected erythrocyte surface was scored as mean fluorescent intensity using the method of Williams and Newbold (2003). In detail, mean fluorescence of parasite-infected erythrocytes was determined using the formula:

(d-c)-(b-a)

*a* = the mean fluorescence intensity (MFI) of uninfected erythrocytes following incubation in negative control plasma; *b* = MFI of parasitised erythrocytes following incubation in negative control plasma; *c* = MFI of uninfected cells incubated in immune plasma or test antibody and *d* = MFI of parasitised erythrocytes incubated in immune plasma or test antibody.

### 2.5 ELISA

*Plasmodium falciparum* (A4 strain) schizont extract was coated onto wells in PBS according to the methods described by Ndungu et al. (2002). Plates were incubated overnight at 4 °C, after which wells were washed four times in PBS/Tween (PBS/0.05% Tween 20), and blocked for 5 h at room temperature with 1% skimmed milk in PBS/Tween (blocking buffer). Wells were washed again and incubated overnight at 4 °C with 100 μl of test sera (1/1 000 dilution in blocking buffer). Plates were then washed four times and incubated for 3 h at room temperature with 100 μl of horseradish peroxidase (HRP)-conjugated rabbit anti-human IgG (Dako Ltd., Buckinghamshire, UK) at 1/5 000 dilution in blocking buffer before final washing and detection with H_2_O_2_ and *O*-phenylenediamine (Sigma, St. Louis, MO, USA). The reaction was stopped with 25 μl of 2 M H_2_SO_4_ per well and absorbance read at 492 nm.

### 2.6 Statistical analysis

All statistical analysis was performed using Stata8™ (StataCorp. CA, USA). The relationship between the presence of parasites and reactivity to the surface of erythrocytes infected with each isolate was assessed using logistic regression. The outcome in every case was positivity for recognition of each isolate as classified using the cut-off of the mean response from 20 non-exposed donors plus 3 SD Initially the data were explored for additional factors affecting the likelihood of recognising each isolate. In every case these were identified as age (in categories of 6 months duration) and previous exposure to malaria infection (estimated by responses to whole schizont extract, considered as a continuous variable). The likelihood of recognition of each isolate was then compared between those individuals who were parasite-positive and those who were parasite-negative at the time of the cross-sectional survey when the plasma samples were drawn.

To explore the relationship between antibody responses, the presence of microscopically detectable parasites at the time of serum collection and subsequent clinical malaria, a multiple logistic model was performed after identification of significant confounding variables. These were identified as age (categorised as a factor of 6 months duration), and exposure (estimated from responses to whole schizont extract). The outcome variable was at least one episode of malaria as defined previously after censoring the first 30 days of follow-up (to take account of the possibility that the child was in fact experiencing an early or partially treated episode of clinical malaria despite being asymptomatic at the time of cross-sectional sampling). Individuals were categorised according to whether or not they had detectable parasitaemia at cross-sectional survey and whether they were positive or negative for recognition of each isolate. This variable was then factorised and analysed as the main explanatory variable in a multiple logistic regression.

When investigating the likelihood of having a greater number of episodes of clinical malaria, an ordered logistic regression was used. Individuals were scored 0, 1, 2, 3 or 4 according to the number of episodes of malaria they suffered during the 6 month follow-up; the resultant odds ratio (OR) gives the likelihood of being in a higher numbered group (i.e. having two episodes compared with one, three compared with two, etc.,) depending on which structured antibody/parasite group the individual fell into. Again confounding variables of age and exposure were included and the first 30 days of follow-up were censored.

Where differences between two continuous variables were assessed, no assumptions of distribution were made and the Wilcoxon rank sum test was used to calculate significance. Where more than two variables were compared, the Kruskal Wallis test was used. All correlations were performed using Spearman’s rank correlation coefficient.

Kaplan–Meier survival curves were plotted using time in days to first episode as previously defined. Throughout the analysis, *P* values less than 0.05 were considered significant.

## 3 Results

### 3.1 Naturally acquired antibody responses to the surface of *P. falciparum*-infected erythrocytes are associated with increasing age and asymptomatic parasitaemia

**Fig. S1:**
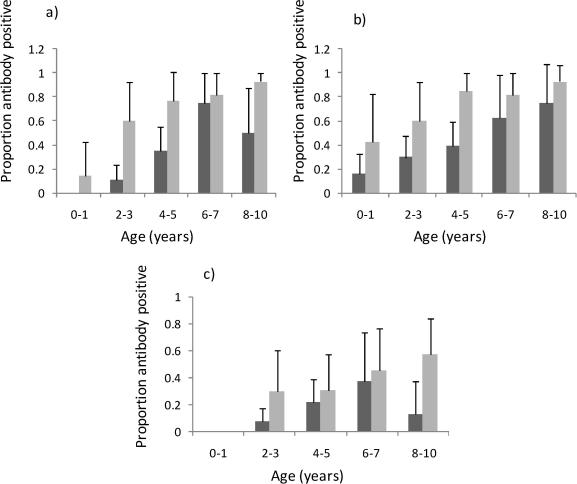
Responses to two laboratory *Plasmodium falciparum* isolates A4 40-cycle and 3D7, and a clinical isolate P1. The proportion of individuals in each age category are shown, with upper 95% confidence interval, scoring positive for antibody recognition of parasite lines A4 40-cycle and 3D7 and clinical isolate P1. Positivity was scored as defined in the text. The dark grey bars represent individuals with no microscopically detectable parasitaemia at the time of the cross-sectional survey and the light grey bars represent individuals with parasites detected at that time. A) responses to A4 40-cycle B) responses to 3D7 C) responses to P1

**Fig. 1:**
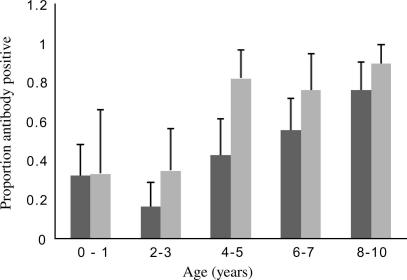
Responses to *Plasmodium falciparum* isolate A4U. The proportion of individuals in each age category is shown, with upper 95% confidence interval, scoring positive for antibody recognition of parasite line A4U. Positivity was scored as defined in the text. The dark grey bars represent individuals with no microscopically detectable parasitaemia at the time of the cross-sectional survey and the light grey bars represent individuals with parasites detected at that time.

Antibody responses to A4U increased significantly with age in individuals who were parasite-positive or parasite-negative at cross-sectional survey (Fig. 1). Responses increased similarly with age for the other parasite isolates used in this study (data not shown, see Supplementary Fig. S1 for details).

However, in those individuals who were parasite-positive at the time of sampling, there was a significantly increased likelihood of recognition of all three laboratory parasite lines and the clinical isolate P1 (A4U OR of recognition if parasite-positive compared with parasite-negative 2.53 (95% CI 1.41–4.54) *P* = 0.002; A4 40-cycle OR 5.05 (95% CI 2.00–12.75), *P* = 0.001; 3D7 OR 4.62 (95% CI 1.92–11.12) *P* = 0.001; P1 OR 2.62 (95% CI 1.01–6.83) *P* = 0.049).

### 3.2 Asymptomatic parasitaemia and risk of clinical malaria

Parasite prevalence at cross-section among children aged 1–10 years in Chonyi was 43.2%. Overall the OR for an individual developing clinical malaria in the 6 months following the cross-sectional survey if they were parasite-positive compared with parasite-negative was 3.42 (95% CI 1.81–6.48; *P* < 0.0001). Limiting the analysis to risks of a clinical attack 30 days after the initial cross-section continued to demonstrate a significant increased likelihood of becoming a case if parasite-positive (OR 2.52 (95% CI 1.21–5.27; *P* = 0.013)).

### 3.3 Association of antibodies to the intact infected-erythrocyte surface and protection from clinical malaria

**Fig. 2:**
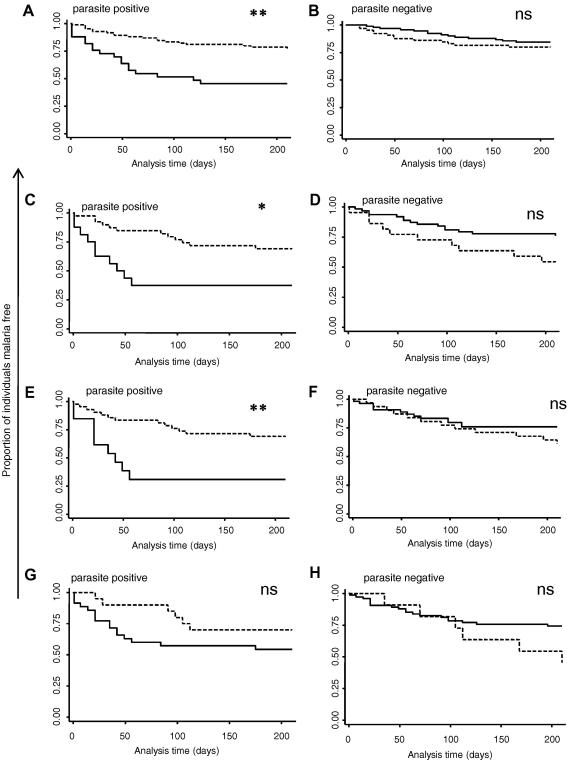
Kaplan–Meier survival curves according to antibody and parasite status. Graphs show the proportion of individuals remaining free from clinical malaria over time (days). Individuals are categorised according to whether they scored positive for antibody recognition of each parasite line in turn (dashed lines) or negative (solid lines)and whether or not they had microscopically detectable parasites at the time of the cross-sectional bleed. (A) and (B) Antibody responses measured against A4U; (C) and (D) antibody responses measured against A4 40-cycle; (E) and (F) antibody responses measured against 3D7; and (G) and (H) antibody responses measured against the clinical isolate P1. ^∗∗^*P* < 0.005 (logrank);^∗^*P* < 0.05 (logrank); ns not significant.

As a result of the interaction between parasite status and antibody recognition of each isolate, all individuals were categorised into four groups according to both their pre-season antibody status (positive or negative) and whether or not they had a microscopically detectable parasitaemia. Kaplan–Meier survival curves were drawn with each of the four groups using responses against each isolate in turn. It is clear that for responses against three of the parasite isolates tested, those individuals with asymptomatic parasite carriage and no concomitant antibody response appeared to be highly susceptible to mild clinical malaria (Fig. 2).

The likelihood of acquiring at least one episode of clinical malaria in the subsequent 6 months was investigated according to group in a multiple logistic model with age and exposure as confounding variables and excluding the first 30 days of follow-up. Table 3 shows the results for responses to all parasites. It is clear that those children with asymptomatic parasitaemia at cross-sectional survey with no concomitant antibody response to A4U had a significantly increased risk of experiencing at least one episode of clinical malaria in the subsequent 6 months (OR = 3.78 (1.21–11.8), *P* = 0.022). The same group was observed to be similarly susceptible when responses to the other three parasite isolates were considered.

When the outcome examined was the total number of malaria episodes experienced by each individual over the follow-up period, an ordered logistic regression model was used with the same confounding variables included and the first 30 days of follow-up excluded. Again the group of children who were antibody-negative and parasite-positive were significantly more likely to experience a greater number of malaria episodes in the subsequent 6 months. This was true for all parasite lines tested (Supplementary Table S1).

**Fig. 3:**
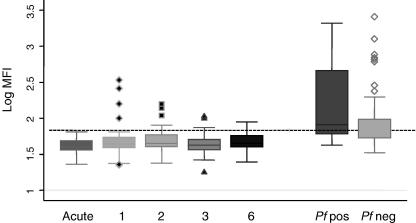
Antibody responses to *Plasmodium falciparum* isolate A4U amongst children presenting to hospital with *P. falciparum* malaria and during follow-up. Shown are the median plus interquartile range of the log transformed mean fluorescence intensity (MFI) for responses to A4U amongst 39 children (mean age 29.5 months) resident in Kilifi District from sera taken at acute presentation (acute) and then at 1, 2, 3 and 6 weeks follow-up. The median and interquartile ranges of the log transformed MFI from asymptomatic parasite-positive children (mean age 29.01 months) and parasite-negative children (mean age 28.96 months) from the same area are shown for comparison. The horizontal line represents the log transformed MFI obtained from 20 non-malaria-exposed UK donors plus 3 SD.

In order to examine antibody responses in a known susceptible group of children, sera from children presenting to hospital with acute malaria were tested for recognition of A4U. Sera were collected at each follow-up visit and again tested for recognition of the surface of unfixed A4U. As can be seen in Fig. 3, children presenting with symptomatic malaria showed no recognition of this highly selected laboratory isolate. Interestingly, they remained unresponsive to this isolate for at least 6 weeks post-presentation. By contrast, children in the same age range, from the same area, with no symptoms of disease, showed significantly greater recognition, whether or not they harboured an asymptomatic parasitaemia (*t*-test *P* < 0.0005 for all comparisons between the community samples and the samples from children presenting to hospital, both acute presentation and all follow-up samples).

### 3.4 Specificity of antibody responses

**Fig. 4:**
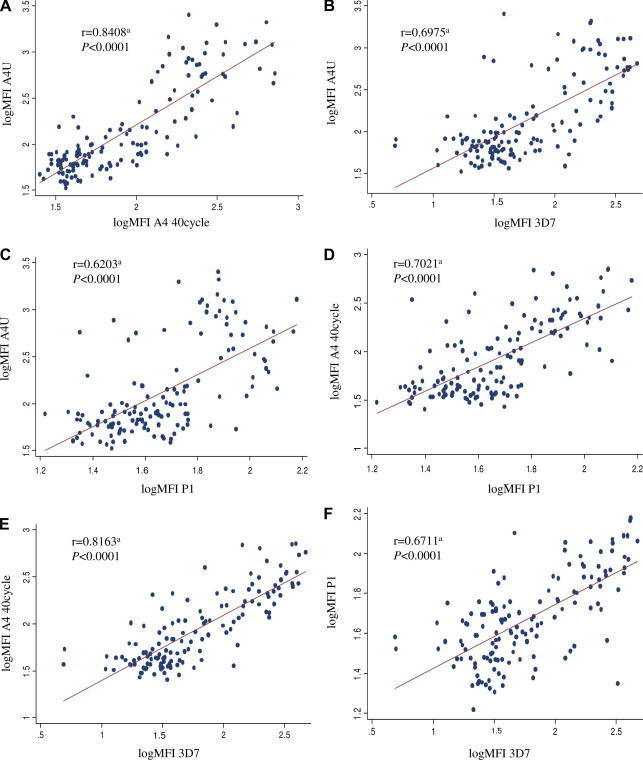
Inter-isolate correlations. Scatter diagrams show correlations of individual responses to pairs of *Plasmodium falciparum* isolates. All mean fluorescence intensity (MFI) scores have been log transformed. (A) A4U versus A4 40-cycle; (B) A4U versus 3D7; (C) A4U versus P1; (D) A4 40-cycle versus P1; (E) A4 40-cycle versus 3D7; (F) P1 versus 3D7. ^a^Spearman’s rank correlation coefficients for each pair of comparisons. Shown are the coefficients and the significance values.

In order to assess the specificity of individual responses scatter diagrams of paired responses, normalised on a log scale, were plotted (Fig. 4). A distinct positive correlation between individual responses to each isolate in turn was observed. This was confirmed by Spearman’s rank correlation coefficient. Furthermore, the possibility that these relationships simply reflected an individual’s exposure to malaria infection was excluded by performing a multiple linear regression model examining the relationship between responses to each isolate controlling for age, parasite status at cross-sectional bleed, and responses to whole schizont extract as a proxy measure of exposure (Supplementary Table S2). In addition, again using a multiple linear regression model, there was no relationship between responses to schizont extract and individual responses to each isolate in turn, further strengthening the premise that exposure alone did not account for the inter-isolate correlations observed (Supplementary Table S3).

## 4 Discussion

While it is accepted that children develop ‘protective’ antibodies specific to the infecting isolate following infection (Bull et al., 1998), it is not clear how relatively cross-reactive antibodies or responses directed against less immunogenic conserved targets on the infected erythrocyte surface are involved in protective immunity.

Previously, associations between antibody responses to isolates not immediately involved in an episode of clinical malaria and subsequent protection have been conflicting (Bull et al., 1998, 2002; Giha et al., 2000; Dodoo et al., 2001). In the Gambia, the best predictor of protection in children was antibodies directed against a randomly selected clinical isolate (Marsh et al., 1989), while in Kenya the ability of sera to agglutinate a randomly selected isolate was not associated with protection (Bull et al., 1998, 2002). In Sudan, antibodies directed against an isolate from Ghana were associated with protection while antibodies to six Sudanese isolates were not. Similarly, in Ghana responses to a Sudanese isolate and to a Ghanaian isolate demonstrated an association with protection but responses to another Ghanaian isolate did not (Giha et al., 2000; Dodoo et al., 2001). These data suggest that responses to some isolates but not others are associated with protection.

We have shown that failure to mount an antibody response against the surface of erythrocytes infected with three laboratory parasite lines, including two phenotypes of a genetically identical isolate, A4 and the reference line 3D7 as well as a locally obtained clinical isolate, was associated with susceptibility to clinical malaria in children with an asymptomatic parasitaemia. The lack of antibody response in children of the same age, from the same area, presenting as a case of acute clinical malaria to hospital, and thus by definition susceptible, supports this claim. It is interesting that up to 6 weeks following successful treatment this group of children did not make a response comparable with those children from the community who were well at the time of serum sampling, thus supporting the possibility that the inability to mount an antibody response in the presence of parasites may be associated with susceptibility.

This increased susceptibility amongst those harbouring parasites but without a concomitant antibody response might reflect a poor inherent ability to respond appropriately in the presence of challenge, as has been suggested in a number of studies (Giha et al., 1999b; Ofori et al., 2002; Kinyanjui et al., 2003). These particular children were also more susceptible to multiple infections within the follow-up period, further strengthening the likelihood that, functionally, they represent a true group of susceptibles.

It has been hypothesised that development of immunity against clinical malaria is associated with the ability to maintain chronic asymptomatic infections (Marsh, 1992; Smith et al., 1993). Chronic infections may predispose a child to a wide range of variant epitopes thus inducing acquisition of a broad repertoire of antibodies. This is supported by modelling work demonstrating that children able to recognise multiple heterologous isolates were more likely to be able to sustain chronic infections (Recker et al., 2004). It may be that the presence of parasites at the time of serum sampling reveals important, short-lived cross-reactive responses and as such allows measurement of the breadth of a child’s antibody repertoire. Interestingly the observation of enhanced anti-erythrocyte surface antibody responses in children with an asymptomatic *P. falciparum* infection, present in many studies (Iqbal et al., 1993; Giha et al., 1999a,b; Bull et al., 2002; Ofori et al., 2002; Osier et al., 2007), and true in this study, was absent in those children who subsequently became a case of malaria. This observation lends support to the premise that the kinetics and effectiveness of the anti-erythrocyte antibody response may be altered in those children who demonstrate susceptibility to clinical malaria.

Of course, the importance of chronic infection in the development of anti-malaria immunity is at odds with the observation in this study that being parasitaemic per se was associated overall with an increased susceptibility to mild clinical malaria. This should be interpreted with caution as it is not an invariable finding and previous surveys in this area have shown no association between asymptomatic parasite carriage at the end of a dry season and protection from, or susceptibility to, severe malarial disease (Bull et al., 2002). It is not clear if these contrasting results reflect different methodologies, different outcome measures of clinical malaria or are due to different levels of exposure amongst the individuals studied. Perhaps most importantly, this apparent susceptibility among parasite-positive individuals can be accounted for almost entirely by the increased incidence of clinical malaria among those who did not mount a concomitant antibody response to any of the parasite lines or isolates tested.

The lack of any association between the presence of anti-erythrocyte surface antibodies and protection from clinical malaria among children without the presence of an asymptomatic microscopically detectable parasitaemia is interesting. It is likely that these antibody responses are short-lived (Ofori et al., 2002; Kinyanjui et al., 2003) and thus the presence of antibodies in the absence of parasites may simply reflect a recently treated acute infection and may be a marker of increased susceptibility to clinical malaria. Although an individual’s prior experience of malaria was taken into account as far as possible by including responses to schizont extract as a variable in the analysis and by excluding the first 30 days of follow-up, it may be that these measures are not comprehensive enough. Furthermore, in those children without parasites at the time of sampling, it is difficult to distinguish those who would make an adequate and broad antibody response if challenged from those genuinely unable to. This issue has become apparent in several recent studies in which analysis of antibody responses within the whole population demonstrated no protective effect but when parasite-positive individuals were analysed separately, a clear protective effect was shown (Kinyanjui et al., 2004b; Polley et al., 2004; Osier et al., 2007).

The apparent lack of isolate specificity of this important antibody response, unlike that acquired following an episode of disease (Bull et al., 1998) suggests that the antigenic targets on the surface of the infected erythrocyte may be relatively conserved. This is supported by the fact that individual responses to each isolate remained highly correlated, even after allowing for exposure as reflected by total anti-schizont response. Whilst this may be expected for comparisons made between A4U and A4 40-cycle, if immune selection results in structuring of *var* genes into discrete antigenic groups (Gupta and Anderson, 1999), it was not expected between the A4 isolates and either 3D7 or the clinical isolate P1.

The biological function of such an antibody response remains highly speculative. There are a number of mechanisms through which antibody directed at the infected erythrocyte surface could mediate protection. While it is known that sera from individuals resident in malaria endemic areas are able to disrupt cytoadherence to various cell-lines in vitro (Udeinya et al., 1983; Singh et al., 1988; Beeson et al., 2004), the relationship of antibodies able to block cytoadherence and protection from clinical disease remains unknown. It is also possible that antibody binding directly to the erythrocyte surface could facilitate parasite clearance through opsonisation and phagocytosis. Again it is clear that sera from individuals in endemic areas have opsonophagocytic activity (Tebo et al., 2002) but there has been no confirmation of any relationship between opsonophagocytic activity and protection from disease in vivo (Marsh et al., 1989).

Identifying both the target for this response and its biological effector mechanism are of considerable importance both for vaccine development and improved understanding of the acquisition of immunity in children in malaria endemic areas.

## Figures and Tables

**Table 1:** Characteristics of individuals tested against *Plasmodium falciparum* isolate A4U

	Age (years)					Total
	0–1	2–3	4–5	6–7	8–10	
Parasite-positive	9	20	28	21	39	117
Parasite-negative	34	36	28	36	21	155
Total	43	56	56	57	60	272

**Table 2:** Randomly selected sub-group of individuals from within original cohort (Table 1) tested against two further *Plasmodium falciparum* parasite lines and one clinical isolate

	Age (years)					Total
	0–1	2–3	4–5	6–7	8–10	
Parasite-positive	7	10	13	11	14	55
Parasite-negative	19	27	23	8	8	85
Total	26	37	36	19	22	140

**Table 3:** Association of anti-erythrocyte surface antibodies and parasitaemia with protection from clinical malaria

	Group^a^			
	ab+ pf+	ab+ pf−	ab− pf+	ab− pf−
Responses against A4U				
*n*	84	65	33	90
% with malaria^b^	22.62	20	54.5	15.6
OR (95% CI)^c^	1.67 (0.60–4.63)	1.35 (0.48–3.78)	3.78 (1.21–11.8)	1
*P*-value	0.325	0.567	0.022	na
				
Responses against A4 40-cycle				
*n*	39	22	16	63
% with malaria^b^	30.8	45.5	62.5	23.8
OR (95% CI)^c^	1.55 (0.55–4.34)	2.88 (0.94–8.82)	5.35 (1.66–17.2)	1
*P*-value	0.402	0.064	0.005	na
				
Responses against 3D7				
*n*	42	31	13	54
% with malaria^b^	30.9	38.7	69.2	24.1
OR (95% CI)^c^	1.43 (0.53-3.91)	2.01 (0.74-5.47)	7.10 (1.87-26.9)	1
* P*-value	0.479	0.170	0.004	na
				
Responses against P1 (clinical isolate)				
* n*	20	11	35	74
% with malaria^b^	30	54.5	45.7	25.7
OR (95% CI)^c^	1.38 (0.42–5.97)	3.72 (0.98–14.1)	2.53 (1.07–5.97)	1
* P*-value	0.587	0.053	0.035	na

a Individuals were stratified according to whether they were antibody-positive (ab+), scored as corrected mean fluorescence intensity (MFI) greater than the mean plus 3 SD of the MFI of 20 non-exposed donors, or negative (ab−) and parasite-positive (pf+) or -negative (pf−) (detected by microscopy) at the time of the cross-sectional survey.

b Malaria defined as at least one episode of fever >37.5 °C plus parasitaemia >2500/μl if aged greater than 1 year and fever >37.5 °C plus any parasitaemia if aged less than 1 year.

c Odds ratio (OR) of becoming a subsequent case of malaria during the 6 month follow-up, obtained from a multiple logistic regression controlling for age (categorised as a factor of 6 month duration) and exposure (estimated from responses to whole schizont extract) and excluding the first 30 days of follow-up.
