# Supplementary material for: Failure to respond to the surface of Plasmodium falciparum infected erythrocytes predicts susceptibility to clinical malaria amongst African children
Source: Int J Parasitol. 2008 Oct;38(12):1445–54. doi: 10.1016/j.ijpara.2008.03.009 (PMC2697313; doi:10.1016/j.ijpara.2008.03.009)
Supplement: Supplementary tables [file mmc1.doc]

**Supplementary Table S1.**

|  | Group**^a^** | | | |
| --- | --- | --- | --- | --- |
|  | ab+ pf+ | ab+ pf- | ab- pf+ | ab- pf- |
| **Responses against A4U** | | | | |
| mean no. malaria  episodes (95% CI)**^b^** | 0.30 (0.17-0.43) | 0.29 (0.13-0.45) | 0.79 (0.49-1.08) | 0.17 (0.08 -0.25) |
| OR (95% CI)**^c^** | 1.52 (0.77-3.47) | 1.54 (0.65-3.64) | 6.65 (2.61-16.97) | 1 |
| *P*-value | 0.318 | 0.330 | 0.022 | na |
| **Responses against A4 40 cycle** | | | | |
| mean no. malaria  episodes (95% CI)**^b^** | 0.31 (0.16-0.46) | 0.55 (0.26-0.83) | 0.94 (0.52-1.36) | 0.37 (0.18-0.55) |
| OR (95% CI)**^c^** | 1.43 (0.55-3.74) | 1.10 (0.42-2.89) | 3.47 (1.34-9.04) | 1 |
| *P*-value | 0.469 | 0.837 | 0.011 | na |
| **Responses against 3D7** | | | | |
| mean no. malaria  episodes (95% CI)**^b^** | 0.33 (0.17-0.49) | 0.48 (0.26-0.72) | 1.0 (0.55-1.45) | 0.37 (0.17-0.57) |
| OR (95% CI)**^c^** | 1.59 (0.53-4.75) | 2.21 (0.78-6.33) | 6.93 (2.06-23.33) | 1 |
| *P*-value | 0.409 | 0.139 | 0.002 | na |
| **Responses against P1 (clinical isolate)** | | | | |
| mean no. malaria  episodes (95% C.I.)**^b^** | 0.30 (0.09-0.51) | 0.54 (0.23-0.86) | 0.66 (0.35-0.90) | 0.39 (0.22-0.56) |
| OR (95% CI)**^c^** | 1.58 (0.44-5.58) | 3.08 (0.88-10.79) | 2.59 (1.09-6.17) | 1 |
| *P*-value | 0.477 | 0.079 | 0.031 | na |

**Odds ratio (OR), by pre-season antibody and parasite status, of experiencing a greater number of malaria episodes.**

^a^Individuals were stratified according to whether they were antibody positive (ab+) (scored as corrected mean fluorescence intensity (MFI) greater than the mean plus 3 S.D. of the MFIof 20 non-exposed donors) or negative (ab-) and parasite positive (pf+) or negative (pf-) (detected by microscopy) at the time of the cross-sectional survey.

^b^Malaria defined as at least one episode of fever > 37.5°C plus parasitaemia > 2,500/µl if aged greater than 1 year and fever > 37.5°C plus any parasitaemia if aged less than 1 year.

^c^Odds ratio (OR) of experiencing a greater number of episodes of malaria during the 6 months follow-up, obtained from an ordered logistic regression controlling for age (categorised as a factor of 6 months duration) and exposure (estimated from responses to whole schizont extract) and excluding the first 30 days of follow-up.

**Supplementary Table S2**

**Relationship between antibody responses to parasite clone A4U and antibody responses to all other parasite lines.**

| **Isolate** | **Coefficient^a^** | **95% CI** | ***P*-value** |
| --- | --- | --- | --- |
| **Univariate analysis with no adjustment for confounding variables** | | | |
| A4 40-cycle  3D7  Clinical isolate P1 | 1.05  0.74  1.38 | 0.94 - 1.17  0.62 - 0.87  1.09 - 1.68 | < 0.001  < 0.001  < 0.001 |
| **Multivariate analysis with adjustment for confounding variables** | | | |
| A4 40-cycle  3D7  Clinical isolate P1 | 1.13  0.71  0.99 | 0.96 - 1.30  0.52 - 0.89  0.64 - 1.33 | < 0.001  < 0.001  < 0.001 |

^a^Coefficient calculated using univariate linear regression. The outcome of interest was the mean fluorescence intensity (MFI) of antibody responses to parasite clone A4U after log transformation. In each case the explanatory variable was MFI of antibody responses to each isolate in turn after log transformation.

^b^Coefficient calculated using multivariate linear regression. The outcome of interest was the MFI of antibody responses to parasite clone A4U after log transformation as above. In each case the explanatory variable of interest was MFI of antibody responses to each isolate in turn after log transformation. The coefficient was adjusted for the effect of age (included as a categorical variable of 6 months duration), parasite status (positive or negative for microscopically detected parasitaemia), and exposure (as estimated by antibody responses to whole schizont extract after log transformation).

**Supplementary Table S3.**

**Relationship between antibody responses to schizont extract and antibody responses to all parasite lines and isolates.**

| **Isolate** | **Coefficient^a^** | **95% CI** | ***P*-value** |
| --- | --- | --- | --- |
| A4U  A4 40-cycle  3D7  Clinical isolate P1 | 0.000051  0.00022  -0.00046  -.000124 | -0.00048-0.00058  -0.00049-0.00093  -0.0014-0.00048  -0.00356-0.003312 | 0.851  0.551  0.334  0.943 |

^a^Coefficient calculated using multivariate linear regression. The outcome of interest was an O.D. of antibody responses to schizont extract, as above. In each case the explanatory variable of interest was the mean fluorescence intensity (MFI) of antibody responses to each isolate in turn. The coefficient was adjusted for the effect of age (included as a categorical variable of 6 months duration), parasite status (positive or negative for microscopically detected parasitaemia).
